# The Genetic Diversity and Population Genetic Structure of the Red Panda, *Ailurus fulgens*, in Zoos in China

**DOI:** 10.3390/ani10061008

**Published:** 2020-06-09

**Authors:** Yun-fang Xiu, Cheng-Chi Liu, Su-hui Xu, Chen-Si Lin, Chin-Cheng Chou

**Affiliations:** 1 Strait (Fuzhou) Giant Panda Research and Exchange Center, No. 88, Mengshan Road, Fuzhou 350001, China; tuna950@gmail.com (Y.-f.X.); lcatc10727255@gmail.com (S.-h.X.); 2 Department of Veterinary Medicine, National Taiwan University, No. 1, Sec. 4, Roosevelt Rd., Taipei 10617, Taiwan; brain.argi@gmail.com (C.-C.L.); cslin100@ntu.edu.tw (C.-S.L.)

**Keywords:** captive red panda, microsatellite, genetic diversity, genetic structure

## Abstract

**Simple Summary:**

Red pandas (*Ailurus fulgens*) have been listed as one of the endangered species in China and worldwide. In order to protect the populations as well as to avoid the inbreeding depression in wildlife, monitoring the genetic diversity and population genetic structure of red pandas is necessary. By analyzing the captive red pandas among the zoos in China using PCR and genotyping, we found a high genetic diversity among the populations as high as that of the wild population. This demonstrate the current scientifically-based breeding program can keep the high genetic diversity in captive red pandas.

**Abstract:**

In China, red pandas (*Ailurus fulgens*) have been raised in zoos for 60 years. It is very important to understand the genetic diversity and population genetic structure of the captive red pandas. Based on 19 microsatellite loci, we investigated genetic diversity and population genetic structure of 116 captive red pandas, with samples taken from 11 captive populations in China. Our results revealed a high genetic diversity among the populations, with mean allelic richness varying from 3.505 (Beijing) to 4.026 (Mianning), and expected heterozygosities varying from 0.631 (Huangshan) to 0.782 (Wenling). In particular, significant deviation from Hardy–Weinberg equilibrium was found in populations of Fuzhou and Jiangsu. The genetic differentiation index across all populations was 0.055, indicating a significant genetic differentiation among the 11 populations. These populations could be divided into three genetic clusters using a microsatellite-based Bayesian clustering analysis, which were consistent with the clustering results of wild populations. We conclude that the genetic diversity among captive red pandas is as high as that of the wild population. More attention should be paid to develop a proper and scientifically-based management program to avoid inbreeding and maintain a high genetic diversity in captive red pandas.

## 1. Introduction

The important issues for conservation and management include both the identification of local populations and the origin of wild individuals. This can be done using morphological characteristics or tagging programs. However, genetic information can provide a niche over traditional methods. Understanding the distribution of genetic variation within a species and how it is affected by human activities is essential for the development of effective conservation and management programs. The red panda (*Ailurus* fulgens), a member of the order Carnivora, is an arboreal vegetarian mammal that depends almost mainly on a bamboo diet. The major threats to wild red pandas include habitat loss, fragmentation, and degradation [1]. Red panda population has declined by 50% over last three generations and this decline is still continuing [2]. The protection of wild populations and good management of captive red pandas are believed equally important for their conservation.

The red panda is one of the earth’s living fossils since their ancestors originate more than ten million years ago across Eurasia. Currently red panda populations live sporadically in bamboo forests of India, Himalayan, and Heng-Duan mountains in Nepal, Bhutan, Myanmar, and China [3]. In China, its distribution is mainly found in Sichuan, Yunnan, and Tibet [4]. Analyses based on mitochondria DNA (mtDNA) and microsatellites can help infer levels of genetic variation, inbreeding, and relatedness within a population. Microsatellites are hypervariable loci, and are popular markers for population and conservation genetic researches at the intraspecific status due to their high mutation rates. The application of microsatellite analysis is viewed as an informative, cost effective, and reproducible method in investigating the genetic structure of animals including red pandas [3].

The three levels of biodiversity are ecosystem diversity, species diversity, and genetic diversity [5]; genetic diversity is the basis of other two counterparts. Comprehensively understanding the genetic diversity of a species facilitates the development of effective conservation strategies and measures. It was reported that in 2015, around 1382 red pandas have been registered with 413 bred in 50 zoos in China. These animals have been raised in China for >60 years [6] and their genetic diversity must be urgently assessed. Previous studies combined nine microsatellite loci and 551 bp of mitochondrial DNA control regions to analyze the genetic structures of wild red pandas across five populations (namely Qionglai, Liangshan, Xiaoxiangling, Gaoligong, and Tibet) [7]. Liang [8] used 28 microsatellite loci to examine the genetic diversity of the red pandas at the Chengdu Research Base of Giant Panda Breeding, Pixian Farm, and Kunming Zoo and observed high genetic diversity in all three populations. These limited results have potentially revealed the wild populations exhibited higher genetic diversity than the captive populations did, and therefore indicate the significance of breeding management within the zoos.

The current status of the genetic diversity of captive red pandas elsewhere in China remains unknown. The present study investigated the genetically diverse levels of 116 captive pandas using microsatellite analysis across the 11 captive populations. The data shown here shall present whether the current strategy of management and breeding programs in China zoos could maintain the versatile genetic structure of red pandas. 

## 2. Methods

### 2.1. Sampling

All the animal handling and experimental protocols were performed following the red panda (*Ailurus fulgens*) care manual (created by the AZA red panda species survival plan^®^) and were carefully reviewed and approved by the ethics committee of the Straits Giant Panda Research and Exchange Center (FZ) with permission number of FZ-SGPREC-100-EA-091. All experiments were performed in accordance with relevant guidelines and regulations. 

Captive red panda samples (n = 116) were collected from zoos or breeding bases in the following locations: Fuzhou, Wuxi, Shanghai, Beijing, Panyu, Guangzhou, Chongqing, Dazu, Mianning, Huangshan, and Wenling. A total of 100 blood samples, 13 skin samples, and 3 hair samples were collected. The blood samples were stored in EDTA–containing vacuum test tubes at −20 °C. The samples of the skin tissue were taken from the outer edge of the ear after topical disinfection and were stored in 75% ethanol. The hair samples were retrieved from the back or from hair follicles on the tail of red pandas and were stored in 75% ethanol.

### 2.2. DNA Extraction

DNA extraction of the blood, skin, and hair samples was conducted through the sodium dodecyl sulfate and phenol–chloroform methods [9]. DNA quality was tested using 1% agarose gel electrophoresis, and concentration was measured using a NanoDrop 1000 spectrophotometer (Thermal Fisher Scientific, Waltham, MA, USA). Subsequently, the solution was diluted to 100 ng/μL and stored at −20 °C.

### 2.3. Polymerase Chain Reaction and Genotyping

According to the previous studies [10,11,12], 19 microsatellite loci were screened for genetic diversity analysis, as shown in Table 1. For developing the multiplex PCR reaction, the forward primers (5′-end) were modified with the following fluorescent dyes: fluorescein amidite (FAM), hexachlorofluorescein (HEX), tetramethylrhodamine (TAMRA), and fluorescein isothiocyanate (FITC). The specifications of the polymerase chain reaction (PCR) are as follows: 2 µL 10 × Buffer (25 mmol/L MgCl_2_), 0.4 µL 2.5 mmol/L deoxyribose-containing nucleoside triphosphates, 10 pmol/L forward and reverse primers (1 µL each), 0.2 µL *Taq* polymerase, 100 ng/uL DNA template of 1.0 µL, and the rest was filled with ultrapure water to 20 µL. PCR amplification steps were as follows: initial denaturation at 95 °C for 5 min, denaturation at 94 °C for 40 s, annealing at 51–60 °C (depending on the primer melting temperature) for 40 s, extension at 72 °C for 40 s. After 35 cycles, the DNA samples were further extended at 72 °C for 10 min and stored at 4 °C thereafter. Subsequently, the PCR products were sequenced using an Applied Biosystems 3730xl DNA Analyzer (Thermal Fisher Scientific, Waltham, MA, USA). The genotyping parameters were configured using GeneMapper 4.0 (Thermal Fisher Scientific, Waltham, MA, USA) and the GeneScan 500 LIZ Size Standard (Thermal Fisher Scientific, Waltham, MA, USA).

### 2.4. Statistical Method

The Microsatellite Toolkit (http://dscar.gene.ie-tcd./microsatellitetoolkit/m) was installed in Excel as an add-on to verify the accuracy of the input allele values and fragment intervals before the polymorphism information content (PIC) was calculated. The number of alleles (*NA*), observed heterozygosity (*H*_O_), expected heterozygosity (*H*_E_), and genetic distance between pairs (*F*_ST_) were calculated using Arlequin 3.5 [13]. FSTAT 2.9.3 [14] was employed for assessing the allelic richness (*AR*) and the fixation indices of F-statistics comprising the inbreeding coefficient of an individual relative to the total population (*F*_IT_), the genetic differentiation index (*F*_ST_), and the inbreeding coefficient of an individual relative to the subpopulation (*F*_IS_). The Hardy–Weinberg equilibrium was tested using GENEPOP 4.0 [15]. The common microsatellite loci between the present study and Liang [8] were used to compare the expected heterozygosity between the two studies through a one-way ANOVA using SPSS 14.0 (SPSS Inc., Chicago, IL, USA). The result of present study also compared the genetic diversity of captive populations with Hu’s study about wild red pandas across the following five populations: Qionglai (QL), Liangshan (LS), Xiaoxiangling (XXL), Gaoligong (GLG), and Tibet (TIB) [7].

Genetic clustering analysis was conducted using Structure 2.3.1 [16]. Ten independent runs were simulated for each value of *K* (the number of clusters) between 1 and 10. The assumed mixed model and the frequency of associated alleles were simulated 1,000,000 times with 100,000 burn-in runs. The number of genetic clusters (*ΔK*) was estimated using the equation *ΔK* = m|L(K + 1) − 2L(K) + L(K − 1)|/s[L(K)] [17].

## 3. Results

### 3.1. Genetic Diversity of Populations

A total of 175 alleles were detected among the 116 red pandas screened through the 19 loci. The number of alleles in each locus ranged between 5 and 18 with a mean value of 9.21 ± 3.6 alleles; *H*_E_ ranged between 0.528 and 0.901, with a mean value of 0.730 ± 0.115. *H*_O_ ranged between 0.513 and 0.926, with a mean value of 0.719 ± 0.145, and *PIC* ranged between 0.469 and 0.889, with a mean value of 0.708 ± 0.131. Except for the moderate polymorphism level observed on the locus Aifu-39, all other loci exceeded 0.5 and exhibited a high polymorphism level, as shown in Table 2.

The expected heterozygosity of the captive populations in this study was compared with that in Liang’s study [8] using the 16 common microsatellite loci. The expected heterozygosity among each captive population was nonsignificant (*p* > 0.05), as shown in Table 3. This result implies these red pandas in the zoos still possess similar level of genetic diversity as the wild animals do. 

The genetic diversity of the captive populations was compared with that of the wild red panda populations in Hu et al. [7]. The different result revealed that the captive populations exhibited a slightly higher genetic diversity than the wild populations did, as shown in Table 4. Among the 11 captive populations, the Fuzhou population had the highest number of mean alleles with 7.11; the Mianning population had the highest allelic richness with 4.026. All 11 captive populations had HE and HO exceeding 0.5, as shown in Table 5. More data needed to be collected and validated for assessing the possible variances and underlying mechanisms.

### 3.2. Hardy–Weinberg Equilibrium Test

The present study verified the Hardy–Weinberg equilibrium of the 11 captive populations. The results revealed that the Fuzhou and Wuxi populations exhibited significant deviations from the Hardy–Weinberg equilibrium (*p* < 0.01), whereas other populations were in equilibrium, as shown in Table 5.

### 3.3. Genetic Differentiation between Pairs of Populations

Table 2 demonstrates that the *F*_IT_ of all loci exhibited the significant value of 0.049 (*p* < 0.01). This result was contributed by 47.36% of the loci, and the *F*_IT_ of all loci ranged from −0.209 (Aifu-12) to 0.269 (Aifu-7).

The mean *F*_IS_ of all loci had a nonsignificant −0.006. Further analysis of the *F*_IS_ of each population revealed that the *F*_IS_ ranged from −0.73 (Beijing) to 0.111 (Huangshan). Only the Wuxi and Huangshan populations had a significantly positive *F*_IS_, indicating low inbreeding levels in most populations, as shown in Table 5.

All loci contributed the significant *F*_ST_ of the corresponding subpopulations (*F*_ST_ = 0.055, *p* < 0.001). Further analysis of the genetic differentiation between pairs of populations revealed that most captive populations exhibited substantial genetic differentiation, as shown in Table 6.

### 3.4. Genetic Structure Analysis

**Figure 1:**
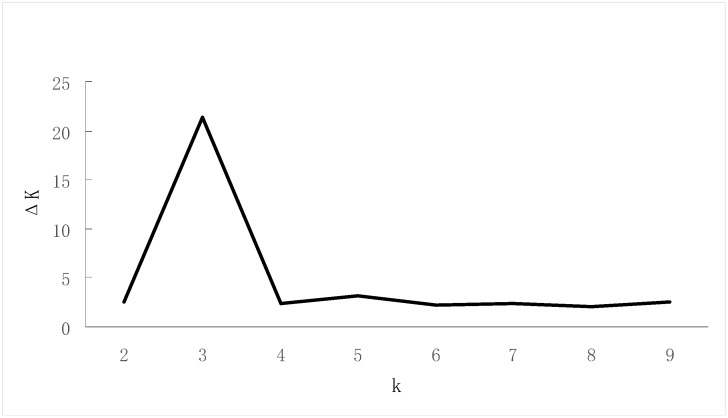
*ΔK* deviation curve.

**Figure 2:**
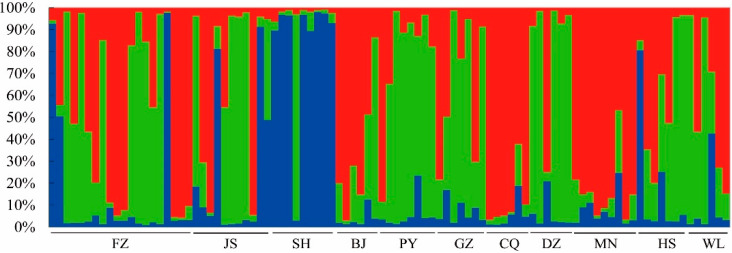
Bayesian genetic clustering of the 11 captive populations.

Genetic structure analysis revealed that the maximum *ΔK* occurred when k = 3 (*ΔK* = 21.4), as shown in Figure 1. Figure 2 illustrates that the 11 captive populations can be grouped into three genetic clusters. When examining the genetic source of each individual in a captive population, the three genetic clusters from other captive populations were identified. In other words, the wild individuals in these captive populations are likely to be associated with several different mountain populations.

## 4. Discussion

Effective genetic management depends on comprehensively understanding the genetic diversity of captive populations. By analyzing the number of alleles, gene polymorphism (PIC), genetic richness, genetic heterozygosity, and the Hardy–Weinberg equilibrium, we revealed the genetic diversity of captive red pandas in the specific zoos in China. 

In conservation genetics research, NA is a critical indicator of the genetic variation of a population. The mean NA of the 11 captive populations ranged from 4.05 (Beijing) to 7.11 (Fuzhou), indicating the abundant genetic variation in captive populations. The mean NA of the Fuzhou population was 7.11, indicating the genetic diversity of this population. This may be attributed to the many red pandas in the Fuzhou population, which increased the NA. Liang et al. [8] reported high NAs from the 28 polymorphic microsatellite loci in the Chengdu Research Base of Giant Panda Breeding (9.09), Pixian Farm (6.69), and Kunming Zoo (3.72), and proposed that all three populations exhibited high genetic diversity. However, sample size is one of the determinants for NA, a larger NA may be detected in a larger sample size. Therefore, a more comparable result can be obtained through the allelic richness of populations. In the study of the effect of habitat cave interference on the population size and genetic richness of David’s myotis (*Myotis davidii*), You et al. [18] reported that allelic richness was not correlated to the population size. Among our 11 populations, the Mianning population exhibited the highest allelic richness at 4.026, whereas the Beijing population had the lowest at 3.505. The analysis of allelic richness indicated that the Mianning population should had a higher genetic diversity.

The comparison of our results and those in the findings from Liang et al. [8] showed that the captive populations had nonsignificant *H*_E_ in 10 loci ranging from 0.668 to 0.779, as shown in Table 3. Hu et al. [7] reported the *H*_O_ and *H*_E_ of 0.679 and 0.719 in the diversity of red pandas in Qionglai, Liangshan, Xiaoxiangling, Gaoligong, and Tibet (n = 105), whereas the present study reported the *H*_O_ and *H*_E_ of 0.719 and 0.730, respectively (n = 116). The mean NA of the wild populations in Hu et al. [7] was 9.2, whereas that of the captive populations here was 9.3, as shown in Table 4. Among the nine microsatellite loci employed in Hu et al. [7], seven were consistent with those in our study. Comparing heterozygosity and mean NA revealed that the captive populations had slightly higher genetic diversity than the wild populations did. However, the meaning of this little difference should need more cases and analysis to uncover the reasons. 

Yan et al. [19] reported that the captive giant pandas in the Wolong National Nature Reserve had a lower genetic diversity than the wild population of giant pandas in Qionglai did, with *H*_E_ of 0.620 and 0.779, respectively. This phenomenon is not observed in red pandas, and we thought that the high genetic diversity of captive red pandas can be attributed to the supplement of wild populations to achieve the genetic admixture of captive and wild individuals. 

Red pandas have a short life expectancy, high mortality rate, and low offspring survival rate, resulting in difficult self-sustainment among the captive populations in China [20]. The sustainability of red panda reproduction relies on supplementary sources from the wild, and some institutions still obtained wild red pandas as of 2007. The high genetic diversity of captive red panda populations can be attributed to the regular infusion of wild blood and manage stud book more efficiently. This necessitates verification of pedigree data and builds a standard management about red pandas in China.

The essence of the Hardy–Weinberg equilibrium in red panda populations refers to the unchanged genetic and genotype frequencies throughout generations in a large-scale and randomized mating population in the absence of external effects such as selection, migration, and mutation. The Hardy–Weinberg equilibrium is based on random mating in natural populations [21]. Endangered species frequently deviate from the Hardy–Weinberg equilibrium because of heterozygote deficiency caused by subspecies, inbreeding, and null alleles [22]. The present study revealed that the captive Fuzhou population significantly deviated from the Hardy–Weinberg equilibrium. Although natural mating was practiced in this population, mating selectivity led to varying breeding participation among individual red pandas. The paternity test of the captive Fuzhou population [23] using microsatellite amplification revealed that two male red pandas were responsible for most of the breeding, which should contribute to the deviation from the Hardy–Weinberg equilibrium.

The present study estimated the genetic status among the red panda populations through an *F*-statistic. The genetic differentiation coefficient (*F*_ST_) is a critical indicator of genetic differentiation among subpopulations. It measures the variance of the average heterozygosity in a subpopulation relative to the total population, ranging between 0 and 1. Wright [24] argued that an *F*_ST_ value between 0 and 0.05 indicates a low level of genetic differentiation between subpopulations, 0.05–0.15 indicates moderate genetic differentiation, and 0.15–0.25 indicates a high level of genetic differentiation. The present study reported a moderate genetic differentiation among the red panda subpopulations in all microsatellite loci (*F*_ST_ = 0.055). Further analysis revealed that most population pairs had a significantly high *F*_ST_, indicating a high level of genetic differentiation between captive populations.

The inbreeding coefficient *F*_IS_ indicated the decreased proportion of heterozygotes because of nonrandom mating among individuals in a subpopulation. The range of *F*_IS_ is between −1 and 1. A positive *F*_IS_ indicates severe inbreeding in a population, resulting in heterozygote deficiency, whereas a negative *F*_IS_ indicates an outbreeding and heterozygote-surplus population. In this study, the captive populations in Fuzhou, Shanghai, Beijing, Guangzhou, and Wenling had negative *F*_IS_ values, indicating a heterozygote surplus; whereas those in Wuxi, Panyu, Chonqing, Dazu, Mianning, and Huangshan had various degrees of heterozygote deficiency. The mean *F*_IS_ of the total population was −0.067, revealing a low level of inbreeding in the total population. Wang et al. [25] examined the genetics of 34 giant pandas in two major captive populations in China (the Chengdu Research Base of Giant Panda Breeding and the China Research and Conservation Center for Giant Panda in Wolong), and reported *F*_IS_ values of 0.3221 and 0.3983, respectively. By contrast, captive red panda populations exhibited lower *F*_IS_, indicating a low level of inbreeding. Inbreeding existed to an extent in some captive populations such as Huangshan (*F*_IS_ = 0.111). The existing captive red pandas have a small population scattered in the zoos throughout China, and few subpopulations have 10 or more red pandas. Therefore, although the existing population has a high level of genetic diversity, the small population may lead to increased inbreeding between captive red pandas in the future.

Cluster analysis is an ideal instrument for genetic structure analysis of a population. The principle of clustering is to assume each analyzed individual has a common ancestor in all categories, and genetically similar individuals are clustered together to estimate the probability values of each category belonging to the said ancestor. When an individual has a more than 80% probability of being classified to a specific category, the individual is inferred to belong in this category. The present study conducted structure analysis to group the 11 captive populations into three clusters. Hu et al. [7] reported that the five wild red panda populations were grouped into three genetic clusters (Gaoligong–Tibet, Xiaoxiangling, and Qionglai–Liangshan). The pedigree record of the Fuzhou captive population revealed that the ancestors originated from (1) Yele County, Mianning, Sichuan (Xiaoxiangling Range), (2) Mabian, Sichuan (Liangshan Range), and (3) Yunnan (Gaoligong Range). The clustering result of the captive Fuzhou population was consistent with that of the wild population, reflecting the mountainous origin of the wild individuals in the captive population. However, most pedigree records of red pandas in China are poorly documented, resulting in difficulties in the development of in-depth genetic management. Therefore, strengthening the investigation of the wild origins of captive red pandas is imperative for the genetic management of captive red panda populations.

## 5. Conclusions

The genetic diversity among captive red pandas is similar to that of the wild population. A proper and scientifically-based management program should be established to avoid inbreeding and maintain the genetic diversity in captive red pandas.

## Figures and Tables

**Table 1:** 19 short tandem repeat (STR) markers used for analysis of genetic diversity.

Loci	Reference	GenBank Accession No.	Primers Sequences (5′–3′)	Repeat Motif	Labelling Dye	Size Range (bp)	Ta (°C)
RP-1	[10]	AY685403	F:CGCCCAGGTACCCTAGAGT	(GT)_5_AT(GT)_10_	FITC	160–198	58
			R:TCCCCACGTTCACTGCAGCATTATC				
RP-11	[10]	AY685406	F:TGAATGTTGCCTTGCTCT	(CA)_12_	FAM	101–127	51
			R:CACCACCTCTTACTGTTCTC				
RP-101	[10]	AY685406	F:ACAGACTGAAAAGGCTTAACAGAGT	(AG)_20_	HEX	155–173	56
			R:CGGTCCATGGTATTCTCCTGTAG				
RP-102	[10]	AY685407	F:ATGCAGAAGAGAATGGAAGCCTGG	(CG)_4_(TG)_10_	HEX	199–209	57
			R:TTCCTGGCAACGATTTCATCCTCAA				
RP-108	[10]	AY60885	F:GTCTCCTCTAACAGCCCACAC	(TG)_3_(CA)_3_CTTA(CA)_16_	TAMRA	217–265	58
			R:GAGGCCACTCTCAACTTTAGTAGAA				
RP-133	[10]	AY685409	F:GCAGGAAGAAGAGGTACTGGTTTCA	(AC)_16_	TAMRA	205–233	56
			R:AACAAGATGCCAGGAAGATACTTTC				
RP-137	[10]	AY685410	F:CACCGTACCCGAGACACCAA	(AC)_16_(AG)_12_	FAM	141–153	52
			R:AAAGAAGAGAAAGTGAAGTGGCAGC				
Aifu-1	[8]	EF408640	F:CCTGCATCAGACTCAGCA	(ATAG)12	FAM	140–176	58
			R:GGTATCAGACGTGGGAACTA				
Aifu-2	[8]	EF408641	F:GACCCAGCCCTAACTCAAA	(CTAC)_11_	HEX	125–141	57
			R:CCTGCATTAGGCTCCACA				
Aifu-5	[8]	EF408644	F:GAATAATGAGCTTGCCTTCC	(GATA)_13_	TAMRA	336–352	55
			R:TTGACATTGGCTATGTGAACA				
Aifu-7	[8]	EF408646	F:CCTGCATCAGACTCAGCA	(GATA)_11_	HEX	221–249	60
			R:CTGGCTTGCAGACAGGAAAT				
Aifu-8	[8]	EF408647	F:TTTACAAAGCAGAGCGTC	(GATA)_15_	FAM	123–171	59
			R:AAATCCTGTCGAACCATG				
Aifu-25	[8]	EF408651	F:AATTGCATGAGCCAGTTC	(CTAT)15	HEX	153–205	54
			R:GCCAGGGTTTTACCAGAG				
Aifu-27	[8]	EF408655	F:CTCAGAATCTTTCATTGCCA	(CTAT)13CCACA(GT)15	TAMRA	255–289	54
			R:TTCCTCAATCCTCTGTTCAA				
Aifu-12	[11]	EF620036	F:TTTGCCAGTAAGCACCCG	(CA)_19_	TAMRA	259–269	58
			R:TGGAACTCAGAGGAGCAGTC				
Aifu-15	[11]	EF620039	F:AGTACCAGCAGTAGGATAGCA	(CA)_3_CG(CA)_6_	FAM	120–150	58
			R:TAAGAGCACTCAGAGGGAAAC				
Rp6	[12]	EU487250	F:ACTGAGGCGAAGAAAGGAGC	(CA)_13_	HEX	154–166	56
			R:CATGGGCATTGAAGATGGTG				
Rp9	[12]	EU487208	F:ACAAACTGGAATGTAAAGG	(CA)_20_	TAMRA	125–143	58
			R:CACATGCTTATGTTATAGGG				
RP13	[12]	EU487212	F:TCCCTTACGCTTCCTCCTTT	(GT)_18_	HEX	126–224	52
			R:GCAGGCGGAGAATTGGTTGG				

**Table 2:** The genetic characteristics of 19 STR from all the samples. * Significant (*p* < 0.05); ** (*p* < 0.01).

Loci	Number of Alleles (*NA*)	Expected Heterozygosity (*H_E_*)	Observed Heterozygosity (*Ho*)	Polymorphism Information Content (*PIC*)	Fixation Indices of the Total Population (*F*_IT_)	Genetic Differentiation Index (*F*_ST_)	Inbreeding Coefficient within the Subpopulation (*F*_IS_)
RP-1	11	0.568	0.547	0.553	0.019	0.062 **	−0.046
RP-101	8	0.853	0.866	0.829	−0.018	0.037 **	−0.057
RP-102	5	0.659	0.600	0.606	0.099 *	0.09 **	0.011
RP-108	13	0.852	0.703	0.837	0.138 **	0.032 **	0.110 **
RP-133	12	0.814	0.844	0.787	−0.028	0.055 **	−0.086
RP-137	7	0.713	0.595	0.676	0.155 **	0.105 **	0.058
Aifu-25	10	0.769	0.786	0.739	−0.024	0.037 **	−0.064
Aifu-27	18	0.901	0.926	0.889	−0.024	0.031 **	−0.056
Aifu-39	6	0.528	0.513	0.469	0.015	0.038 **	−0.026
Aifu-1	9	0.827	0.867	0.848	0.049 *	0.035 **	0.015
Aifu-2	5	0.571	0.525	0.515	0.120 *	0.115 **	0.005
Aifu-5	5	0.732	0.762	0.681	−0.046	0.033 *	−0.082
Aifu-7	11	0.776	0.519	0.762	0.269 **	0.067 **	0.217 **
Aifu-8	12	0.854	0.835	0.845	0.038	0.056 **	−0.018
Aifu-12	6	0.730	0.875	0.681	−0.209	0.04 **	−0.259
Aifu-15	7	0.661	0.602	0.599	0.111	0.034 *	0.08
Rp6	6	0.601	0.625	0.531	−0.075	0.03 **	−0.108
Rp9	10	0.779	0.867	0.751	0.115 **	0.091 **	0.027
RP11	14	0.878	0.809	0.856	0.097 *	0.067 **	0.032
All loci	175				0.049 **	0.055 **	−0.006
Mean ± SD	9.210 ± 3.6	0.730 ± 0.115	0.719 ± 0.145	0.708 ± 0.131			

**Table 3:** The comparison of mean expected heterozygosity from captive populations. * Source: Liang (2007).

Locus	* Chengdu	* Pixian	* Kunming	Fuzhou	Wuxi	Shanghai	Beijing	Panyu	Guangzhou	Chongqi	Dazu	Mianning	Huangshan	Wenling
Aifu-1	0.852	0.811	0.786	0.878	0.865	0.875	0.727	0.833	0.769	0.773	0.833	0.765	0.857	0.833
Aifu-2	0.720	0.742	0.464	0.408	0.697	0.739	0.439	0.625	0.560	0.409	0.697	0.451	0.495	0.621
Aifu-5	0.787	0.721	0.250	0.688	0.783	0.654	0.714	0.767	0.703	0.689	0.750	0.717	0.833	0.712
Aifu-7	0.606	0.774	0.679	0.805	0.662	0.882	0.511	0.517	0.747	0.682	0.429	0.846	0.788	0.733
Aifu-8	0.833	0.774	0.893	0.864	0.805	0.556	0.818	0.858	0.857	0.712	0.848	0.941	0.846	0.879
Aifu-25	0.803	0.884	0.785	0.784	0.706	0.732	0.712	0.700	0.697	0.667	0.733	0.692	0.758	0.833
Aifu-27	0.874	0.948	0.893	0.885	0.784	0.955	0.864	0.875	0.933	0.879	0.848	0.950	0.955	0.844
Aifu-12	0.862	0.816	0.750	0.763	0.827	0.582	0.682	0.658	0.626	0.758	0.644	0.642	0.511	0.712
Aifu-15	0.667	0.784	0.893	0.585	0.662	0.442	0.803	0.700	0.788	0.644	0.758	0.660	0.538	0.803
Aifu-39	0.636	0.537	0.857	0.508	0.500	0.621	0.409	0.517	0.143	0.318	0.591	0.621	0.582	0.652
Mean	0.764	0.779	0.725	0.717	0.729	0.704	0.668	0.705	0.682	0.653	0.713	0.728	0.716	0.762

**Table 4:** The comparison of genetic diversity from captive populations and wild populations of red panda. The bold indicates the sum of the above numbers.

Populations	No. of Individuals	MNA	*H*_O_	*H*_E_
Qionglai	26	6.2	0.743	0.688
Liangshan	28	5.8	0.700	0.704
Xiaoxiangling	27	5.8	0.564	0.634
Gaoligong	20	7.1	0.712	0.732
Tibet	4	4.6	0.722	0.770
**The wild population**	**105**	**9.2**	**0.679**	**0.719**
Fuzhou	41	7.1	0.732	0.722
Wuxi	11	5.2	0.711	0.647
Shanghai	9	5.0	0.693	0.747
Mianning	9	4.0	0.715	0.734
Panyu	8	5.0	0.693	0.746
Guangzhou	7	4.5	0.669	0.684
Huangshan	7	3.8	0.702	0.631
Beijing	6	4.1	0.683	0.744
Chongqi	6	4.2	0.673	0.644
Dazu	6	3.9	0.749	0.734
Wenling	6	3.9	0.740	0.782
**The captive population**	**116**	**9.3**	**0.719**	**0.730**

**Table 5:** Genetic diversity from 11 captive red panda populations. * Significant (*p* < 0.05).

Population	Number of Individual	Mean Number of Alleles (MNA)	Mean Allelic Richness (MAR)	Expected Heterozygosity (*H_E_*)	Observed Heterozygosity (*Ho*)	Inbreeding Coefficient within the Subpopulation (*F*_IS_)	Hardy-Weinberg Equilibrium (*p*)
Fuzhou	41	7.11	3.913	0.722	0.732	−0.033	0.0005 *
Wuxi	11	5.21	3.751	0.647	0.711	0.088 ^*^	0.0019 *
Shanghai	9	5.00	3.767	0.747	0.693	−0.083	0.5411
Beijing	6	4.05	3.505	0.744	0.683	−0.173 ^*^	0.7836
Panyu	8	4.95	3.837	0.746	0.693	0.093	0.1594
Guangzhou	7	4.53	3.748	0.684	0.669	−0.013	0.7825
Chongqi	6	4.16	3.573	0.644	0.673	0.062	0.5984
Dazu	6	4.37	3.908	0.734	0.749	0.015	0.4263
Mianning	9	5.42	4.026	0.728	0.715	0.001	0.5719
Huangshan	7	4.58	3.797	0.631	0.702	0.111 ^*^	0.5751
Wenling	6	4.53	3.903	0.782	0.740	−0.067	0.5476

**Table 6:** Genetic differentiation between pairs of red panda populations (*F*_ST_). * Significant (*p* < 0.05); ** (*p* < 0.01).

	Fuzhou	Wuxi	Shanghai	Beijing	Panyu	Guangzhou	Chongqi	Dazu	Mianning	Huangshan
Fuzhou										
Wuxi	0.03516 ^**^									
Shanghai	0.09036 ^**^	0.06169 ^**^								
Beijing	0.09087 ^**^	0.08553 ^**^	0.14506 ^**^							
Panyu	0.04102 ^**^	0.01701	0.08110 ^**^	0.06893 ^**^						
Guangzhou	0.05748 ^**^	0.01548	0.09509 ^**^	0.14693 ^**^	0.02757					
Chongqi	0.06221 ^**^	0.08104 ^**^	0.15260^**^	0.08600 ^*^	0.07274 ^**^	0.13591 ^**^				
Dazu	0.05353 ^**^	0.02749 ^**^	0.06415 ^**^	0.06820 ^*^	−0.00950	0.08728 ^*^	0.08683 ^**^			
Mianning	0.03928 ^**^	0.05463 ^**^	0.10666 ^**^	0.11472 ^**^	0.03229 ^*^	0.06161 ^**^	0.08486 ^**^	0.33040		
Huangshan	0.02033 ^*^	−0.00260	0.08273 ^**^	0.09260 ^**^	0.02300	0.03790 *	0.05496 ^*^	0.03520	0.04779 ^**^	
Wenling	0.02254	−0.01230	0.06954 ^**^	0.06412 ^*^	−0.01610	0.00955	0.04418	0.01600	0.03280 ^*^	−0.00860
